# Patrolling monocytes inhibit osteosarcoma metastasis to the lung

**DOI:** 10.18632/aging.104041

**Published:** 2020-11-16

**Authors:** Ting Chen, Lin Zhao

**Affiliations:** 1 Department of Pharmacology, School of Pharmacy, Liaoning Key Laboratory of Molecular Targeted Anti-Tumor Drug Development and Evaluation, Liaoning Cancer Immune Peptide Drug Engineering Technology Research Center, Key Laboratory of Precision Diagnosis and Treatment of Gastrointestinal Tumors, Ministry of Education, China Medical University, Shenyang, Liaoning Province, China; 2 Department of Orthopedics, Shengjing Hospital of China Medical University, Shenyang, Liaoning Province, China

**Keywords:** osteosarcoma, patrolling monocytes, metastasis, tumor-infiltrating immune cells, nomogram

## Abstract

Immune infiltration is associated with osteosarcoma metastasis. However, previous studies have not accounted for the functional diversity of the cells involved in the immune response. We conducted a comprehensive comparative analysis of the tumor-infiltrating immune cells in metastatic and non-metastatic osteosarcoma tissues based on a deconvolution algorithm (CIBERSORT). Twenty-two immune cell subsets were evaluated for their association with the presence or absence of metastasis in osteosarcoma patients. A lack of monocytes was associated with osteosarcoma metastasis; however, the levels of M1 macrophages, M2 macrophages and other immune cell subsets did not differ between the metastatic and non-metastatic groups. Additionally, a higher proportion of monocytes was associated with a better prognosis in osteosarcoma patients. Animal experiments demonstrated that the number of metastatic nodules was higher in mice lacking patrolling monocytes than in control mice. Our data indicated that the cellular composition of the immune infiltrate may subtly differ among osteosarcoma patients, and that patrolling monocytes inhibit osteosarcoma metastasis to the lungs of mice. Thus, the composition of the immune infiltrate and the level of patrolling monocytes may be important determinants of whether metastasis occurs in osteosarcoma patients.

## INTRODUCTION

Osteosarcoma is the most common type of primary malignant bone tumor in children and young adults [1]. Despite the availability of treatments such as surgery and chemotherapy, the five-year survival rate is less than 70% and has not improved much in the past few decades [2, 3]. In patients with metastatic osteosarcoma, the prognosis is particularly dismal, as the five-year survival rate is less than 30%. About 50% of osteosarcoma patients develop fatal lung metastases in the later stages of the disease [4]. In developed countries, 95% of cancer-related deaths are caused by metastases. Therefore, new diagnostic and therapeutic methods are urgently needed to improve the prognoses of these patients.

Although the study of lung metastasis-specific biomarkers for osteosarcoma has expanded over the years, the clinical applications of this research are still limited. More recently, studies employing next-generation sequencing and other screening technologies have identified a series of promising biomarkers with predictive and diagnostic value for osteosarcoma-associated lung metastasis [5]. However, many studies have demonstrated that cancer growth and metastasis are determined not only by the intrinsic activities of the tumor, but also by the immune cells in the tumor microenvironment [6, 7, 8]. For example, tumor-associated macrophages promote tumor progression to varying degrees by cultivating cancer stem cells, promoting genetic instability, supporting metastasis and suppressing protective adaptive immunity [9]. Additionally, inflammatory cells and tissues guide the formation of the blood vessels that nourish growing tumors [10, 11]. Thus, the immune system is important for both carcinogenesis and tumor angiogenesis [12, 13]. The functional components of tumor-infiltrating immune cells (TIICs) change subtly with changes in the host immune system, and these cells have been reported to be associated with the clinical prognosis of cancer patients [14]. TIICs exhibit complex interactions with malignant cells in the tumor stroma [15]. Because the immune system supports both host defense and tumor progression, it is a key determinant of prognosis.

Osteosarcoma is an immunosensitive tumor type that is infiltrated by macrophages, monocytes, dendritic cells, neutrophils, mast cells and other heterogeneous cell types, which have high prognostic value according to their type, density and location [9, 16, 17]. In previous studies, the immune cell compositions of tumors have been assessed using immunohistochemistry and flow cytometry, but these technologies have limitations. Immunohistochemistry can only be used to evaluate a few immune cell types at the same time, and flow cytometry usually depends on multiple markers to define certain cell types, but the proteins are limited by the number of fluorescent channels. Therefore, no studies have elucidated the prognostic value of different TIIC subgroups in osteosarcoma.

The biological tool CIBERSORT (Cell-type Identification By Estimating Relative Subsets Of RNA Transcripts) extensively deconvolutes gene expression data and uses complex algorithms to quantify many immune cell types in heterogeneous samples [18]. Therefore, this tool can effectively determine the diversity and landscape of TIICs. In this study, we used CIBERSORT to quantify 22 TIIC subsets in osteosarcoma, in order to explore their relationship to metastasis and survival. Our data have revealed the association between intratumoral immune cell heterogeneity and osteosarcoma progression.

## RESULTS

### Distribution of TIICs in metastatic and non-metastatic osteosarcoma tissues

**Figure 1:**
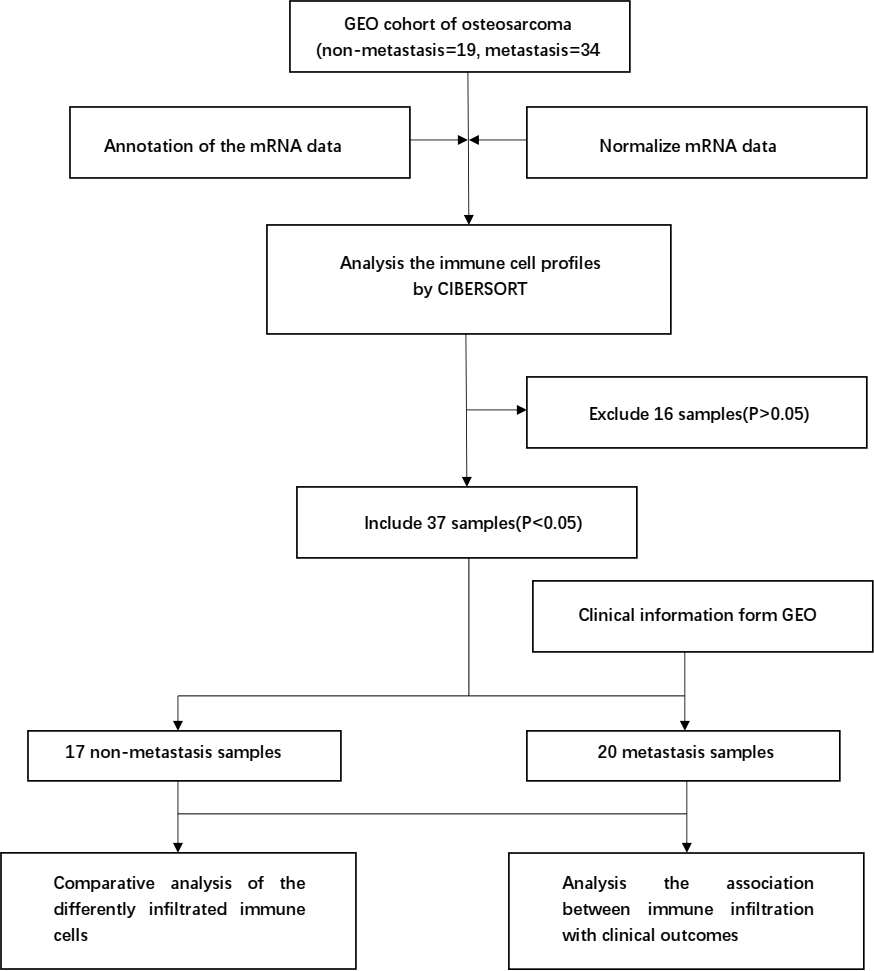
**Flowchart detailing the study design and samples at each stage of analysis.**

**Figure 2:**
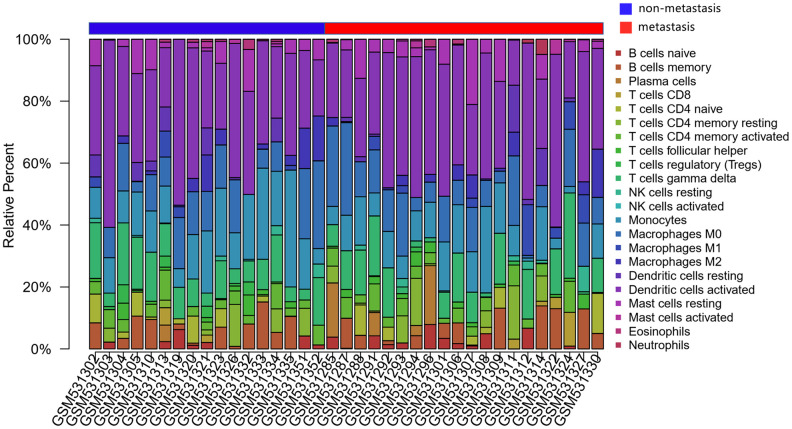
**The landscape of immune infiltration in osteosarcoma.** The differences in immune infiltration between non-metastatic and metastatic tissues are shown.

**Figure 3:**
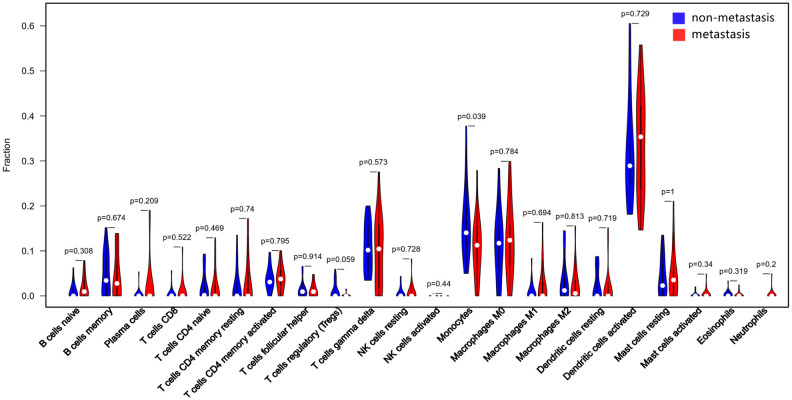
**Quantification of the differing TIIC subtype distributions between non-metastatic and metastatic tissues.** The results were generated using the R software package vioplot.

The CIBERSORT algorithm was used to investigate the differences in 22 subgroups of TIICs in metastatic and non-metastatic osteosarcoma samples. The flow diagram of our protocol and sample enrollment process is shown in Figure 1. In total, 17 non-metastatic tissues and 20 metastatic tissues met the inclusion criteria (P<0.05). The levels of the different TIICs in the metastatic and non-metastatic osteosarcoma groups are shown in Figure 2, and the fraction of immune cells is shown in Supplementary Table 1. When we compared the average proportion of each immune cell type between the metastatic and non-metastatic groups, we found that monocytes were more abundant in the non-metastatic group (P=0.039, Figure 3).

### Correlations between pairs of immune cells

**Figure 4:**
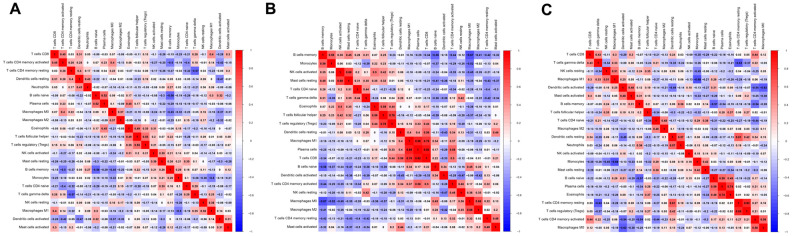
**Correlation matrix of all 22 immune cell densities in different groups.** The results are shown for all tissues (**A**), non-metastatic tissues (**B**) and metastatic tissues (**C**). Red indicates a positive correlation, while blue indicates a negative correlation; the darker the color, the stronger the correlation. The results were generated using the R software package corrplot.

Next, we evaluated the correlations between different pairs of TIICs in osteosarcoma tissues to determine the relationships of various infiltrating immune cells to one another. Figure 4 displays the R value for each pair of immune cells in all patients, as well as in the non-metastatic and metastatic groups. Positive correlations are shown in red, while negative correlations are shown in blue. In all patients, regulatory T (‘Treg’) cell levels and follicular helper T cell levels exhibited the closest correlation, with an R value of 0.55. In contrast, there was a negative association between resting CD4^+^ memory T cell levels and γδ T cell levels (R=-0.57). In the non-metastatic group, CD8^+^ T cell levels correlated strongly with plasma cell levels (R=0.92), and plasma cell levels were positively associated with M1 macrophage levels (R=0.86), whereas memory B cell levels were negatively associated with M0 macrophage levels (R=-0.67). In the metastatic group, Treg cell levels and resting CD4^+^ memory T cell levels displayed the closest correlation, with an R value of 0.83. On the other hand, monocyte levels were negatively associated with M1 macrophage levels (R=-0.68). These results suggested that differences in the 22 subgroups of TIICs between metastatic and non-metastatic osteosarcoma tissues may be clinically significant.

### Association of immune infiltration with prognosis in osteosarcoma patients

**Figure 5:**
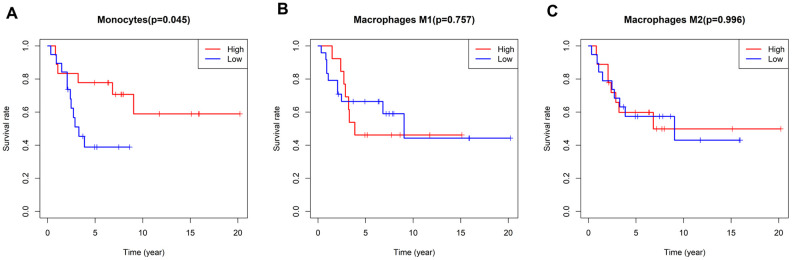
**Survival plots based on the median proportions of specific immune cell subsets.** (**A**) Kaplan-Meier curves showing the different survival rates in patients with high and low-densities of monocytes. (**B**, **C**) There were no differences in patients’ survival times according to the levels of M1 and M2 macrophages. The results were generated using the R software package survival.

As mentioned above, the five-year survival rate of patients with metastatic osteosarcoma is extremely low, so new diagnostic and treatment methods are urgently needed to improve the prognoses of these patients. We analyzed the correlation between immune infiltration and survival time in the enrolled osteosarcoma patients, and found that a higher proportion of monocytes was associated with a better prognosis (P=0.045) (Figure 5). There were no differences in survival time according to the levels of the other TIICs.

### Immune clusters associated with the effectiveness of chemotherapy in osteosarcoma patients

**Figure 6:**
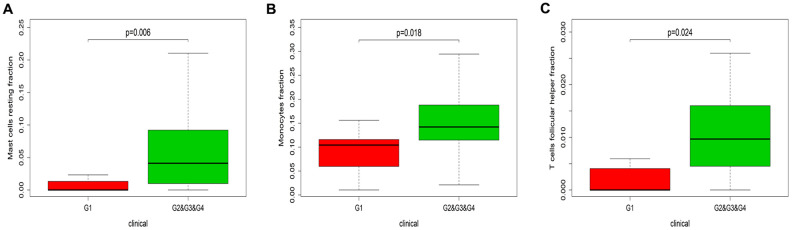
**Immune infiltration in patients with different Huvos necrosis grades.** The results are shown for resting mast cells (**A**), monocytes (**B**) and follicular helper T cells (**C**). G1, chemotherapy is invalid; G2&G3&G4, chemotherapy is effective. The P values were obtained using the Wilcoxon signed rank test in R software.

Next, we designed a nomogram based on the Huvos necrosis grade and the TIIC density to assess the relationship between TIICs and the effectiveness of chemotherapy. The fractions of resting mast cells (P=0.006), follicular helper T cells (P=0.018) and monocytes (P=0.024) were higher in patients with Huvos necrosis grades of 2, 3 and 4 than in those with a Huvos necrosis grade of 1 (Figure 6). These results indicated that higher fractions of resting mast cells, follicular helper T cells and monocytes were associated with greater chemotherapeutic effectiveness.

### Patrolling monocytes (PMOs) inhibited the metastasis of osteosarcoma cells *in vivo*

**Figure 7:**
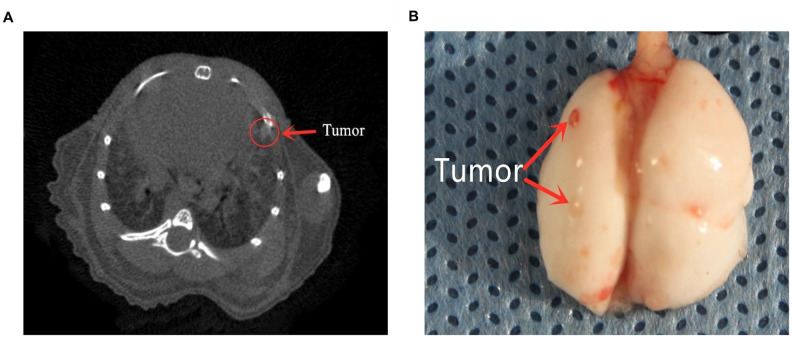
**The formation of lung metastases.** (**A**) Representative images of micro-CT scans. Metastatic tumors can be seen. (**B**) Lung photograph from one mouse. Red arrows indicate metastatic nodules.

**Figure 8:**
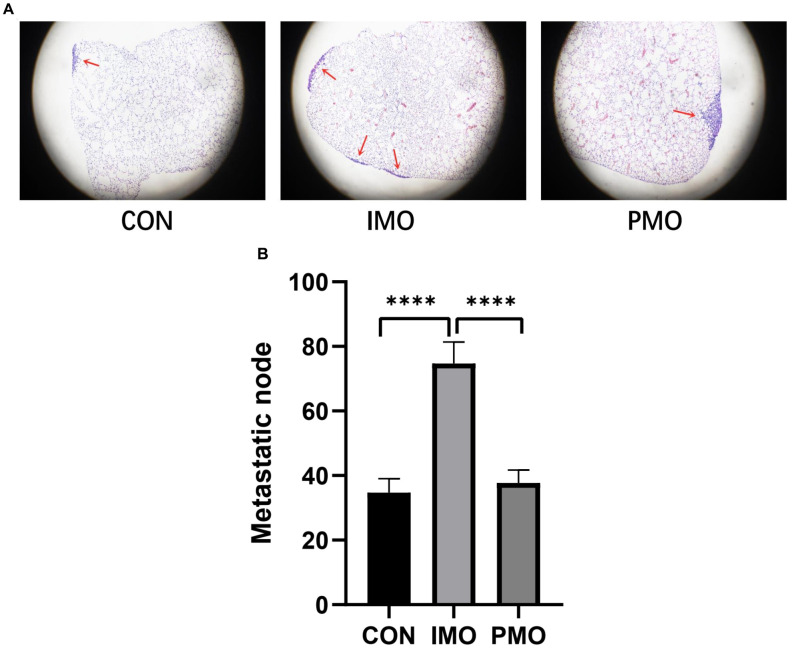
**PMOs inhibited the metastasis of osteosarcoma.** K7M2 WT cells were injected into the tail veins of mice in the control, IMO and PMO groups, and metastatic nodules in the lungs were quantified. (**A**) H&E staining of lung tissues from each group. Representative images are shown. (**B**) The number of metastatic nodules was counted. ****, P<0.0001 in an unpaired two-tailed t test.

To verify the association between monocyte levels and osteosarcoma metastasis, we evaluated the lungs of three different groups of mice injected with K7M2 wild-type (WT) osteosarcoma cells. The control group was injected with phosphate buffered saline-loaded liposomes three days before the K7M2 WT transplant; the inflammatory monocyte (‘IMO’) group was injected with clodronate-loaded liposomes three days before the K7M2 WT transplant, and thus lacked PMOs; and the PMO group was injected with clodronate-loaded liposomes seven days before the K7M2 WT transplant, and thus exhibited normal PMO levels. Nine days after the K7M2 WT transplant, lung micro-computed tomography (micro-CT) was performed to confirm the formation of lung metastases (Figure 7A), and metastatic nodules in the lungs were photographed (Figure 7B). The extent of tumor metastasis was also evaluated via hematoxylin and eosin (H&E) staining of paraffin-embedded lung tissues (Supplementary Figure 1). Histologic examination of micro-metastatic lung lesions suggested that the IMO group had more metastatic nodules than the control and PMO groups; however, there was no difference between the control group and the PMO group (Figure 8A, 8B).

## DISCUSSION

Although more and more studies have identified biomarkers to predict osteosarcoma-derived lung metastasis, the clinical applications of these biomarkers have been limited, and screening studies for osteosarcoma patients are still mostly in the experimental stage. TIIC subpopulations such as M1 macrophages, natural killer (NK) cells and memory T cells are usually associated with a good prognosis, while Treg cells and M2 macrophages are associated with a poor prognosis [23–25]. However, due to technical limitations, previous research has had a very narrow view of the immune response, and the distribution and proportion of TIICs has been determined based on too few specific markers.

In this study, we used CIBERSORT to assess whether immune cell infiltration differed between 20 metastatic and 17 non-metastatic osteosarcoma samples. We detected 22 types of TIICs, and found that monocyte levels were higher in non-metastatic than in metastatic samples, suggesting that monocytes may inhibit the metastasis of osteosarcoma. There are at least two types of circulating monocytes in the blood: classical IMOs and nonclassical PMOs. Classical IMOs (CCR2^High^Ly6C^+^ in mice; CCR2^High^ CD14^+^CD16^-^ in humans) promote tumor metastasis upon their recruitment to tumor sites [26, 27]. On the contrary, nonclassical PMOs (CX3CR1^High^Ly6C^-^ in mice; CX3CR1^High^CD14^-^CD16^+^ in humans) have been reported to prevent melanoma metastasis to the lungs by recruiting NK cells [28, 29].

In the second part of this study, we evaluated the correlations between different types of TIICs in osteosarcoma tissues. Different correlations were observed in the metastatic and non-metastatic groups. Gao et al. reported that CD8^+^ T cells and NK cells could inhibit tumor growth and metastasis in mice [30]. Moreover, a lung cancer study indicated that Treg cells promoted tumor metastasis [31]. Our results demonstrated that plasma cells, CD8^+^ T cells, M1 macrophages, NK cells, Treg cells and CD4^+^ memory T cells were crucial determinants of tumor metastasis, consistent with previous reports [30, 32].

When we assessed the association between TIIC levels and the overall survival times of osteosarcoma patients, we found that patients with higher monocyte densities exhibited longer overall survival times. This finding was consistent with our earlier finding that higher monocyte levels inhibited the metastasis of osteosarcoma. We also assessed the efficacy of chemotherapy by histologically analyzing tumor necrosis and grading it according to the Huvos score [33]. We found that higher proportions of resting mast cells, follicular helper T cells and monocytes were associated with better chemotherapeutic effects.

Based on the results of our bioinformatic analysis and previously published studies, we designed animal experiments to determine which subsets of monocytes could inhibit osteosarcoma metastasis. The number of pulmonary metastatic nodules of osteosarcoma was significantly greater in mice lacking PMOs than in those with normal PMO levels. These results demonstrated that PMOs inhibited the pulmonary metastasis of osteosarcoma, consistent with the results of Hanna et al. [11, 29, 34]. However, further research is needed to determine how PMOs recognize tumor cells to prevent their metastasis to the lungs, and whether PMOs directly kill tumor cells.

In conclusion, our analysis of 22 immune cell subsets in osteosarcoma revealed that certain TIICs were significantly associated with clinical outcomes and had the potential to identify patients who could benefit from chemotherapy. We found that PMOs inhibited the pulmonary metastasis of osteosarcoma, and that resting mast cells, follicular helper T cells and monocytes were associated with good chemotherapeutic results.

## MATERIALS AND METHODS

### Data source and preprocessing

The expression data were downloaded from the Gene Expression Omnibus (GEO) database (http://www.ncbi.nlm.nih.gov/geo) [19]. GSE21257, a microarray dataset of 53 pre-chemotherapy biopsies from osteosarcoma patients, and these data were analyzed using the CIBERSORT signature. The patients’ clinical characteristics were also retrieved. Metastases occurred in 34 of the 53 osteosarcoma patients within five years, and did not occur in the other 19 patients. The samples from these 53 patients were included in subsequent analyses.

### Evaluation of TIICs

Normalized gene expression data were used to quantify the relative proportions of 22 types of infiltrating immune cells using the analytical tool CIBERSORT. The 22 cell types identified by CIBERSORT include B cells, T cells, monocytes, NK cells, macrophages, mast cells, dendritic cells, neutrophils and so on. CIBERSORT is a gene expression-based deconvolution algorithm that uses a set of reference gene levels (a “signature matrix” of 547 genes) to determine the composition of immune cells in a sample. The proportions of cell types in tumor samples with mixed cell types can be inferred using support vector regression based on this signature matrix. After the CIBERSORT program was used, the distribution of the 22 types of infiltrating immune cells, the correlation coefficients and the P values were determined.

### Mice and tumor cell line

Four- to eight-week-old female BALB/c mice (Changsheng, Liaoning, China) were bred in pathogen free conditions at the Animal Center of China Medical University. All animal studies were performed in accordance with the requirements of the Animal Research Committee at China Medical University. K7M2 WT cells were purchased from the Cell Bank of the Chinese Academy of Sciences (November 2019). The cells were maintained in Dulbecco’s Modified Eagle’s Medium (HyClone) with 10% fetal bovine serum (HyClone), 100 mg/mL penicillin (Invitrogen) and 100 U/mL streptomycin (Invitrogen) at 37 in a 5% CO_2_ and 95% air incubator.

### *In vivo* depletion of blood monocytes

Mouse peripheral blood monocytes were depleted using clodronate liposomes (Clodronate Liposomes, Amsterdam, Netherlands), as previously described [20–22]. Briefly, clodronate-loaded liposomes (200 μL) were injected into the tail vein of the mouse either three or seven days prior to the transplantation of K7M2 WT cells. Previous studies have demonstrated that monocytes in the peripheral blood completely disappear on the first day after the injection of clodronate liposomes. On the third day, inflammatory Ly6C^high^ monocytes return to normal, while PMOs are still absent, and on the eighth day, patrolling Ly6C^low^ monocytes return to normal. Taking advantage of this effect of clodronate liposomes, we randomly divided the mice into three groups: the control group (injected with phosphate buffered saline-loaded liposomes before the K7M2 WT transplant), the IMO group (injected with clodronate liposomes three days before the K7M2 WT transplant, and thus lacking PMOs), and the PMO group (injected with clodronate liposomes seven days before the K7M2 WT transplant, and thus exhibiting normal PMO levels).

### *In vivo* pulmonary metastasis animal study

K7M2 WT cells (1 x 10^6^) were injected into the tail veins of mice, ten days after a single injection, lung tumorigenesis was assessed using micro-CT. After the mice were euthanized, their lungs were dissected and fixed with formalin. In order to evaluate the extent of tumor metastasis more accurately, we fixed the right lung tissues of the mice, embedded them in paraffin and sectioned them at 100-μm intervals. H&E staining was performed so that metastatic nodes could be observed and counted under a microscope.

### Statistical analyses

The levels of the 22 types of infiltrating immune cells in patients’ samples were assessed with the Wilcoxon signed rank test, and a violin plot was constructed using the R package vioplot (CRAN.R-project.org/package=vioplot). The R package corrplot (CRAN.R-project.org/package=corrplot) was used to evaluate the correlations between pairs of immune cell subsets.

Kaplan Meier curves were used to evaluate the relationship between immune cell infiltration and overall survival in the R package survival (CRAN.R-project.org/package=survival). The Wilcoxon signed rank test was used to evaluate the relationship between the infiltrating immune cell distribution and the chemotherapeutic effectiveness.

All statistical tests were two-sided, and a P value <0.05 was considered statistically significant.

### Data availability

We downloaded the data from a publicly available database: GEO (accession number: GSE21257).

## Supplementary Material

Supplementary Figure 1

Supplementary Table 1
