# Supplementary material for: Patrolling monocytes inhibit osteosarcoma metastasis to the lung
Source: Aging (Albany NY). 2020 Nov 16;12(22):23004–16. doi: 10.18632/aging.104041 (PMC7746373; doi:10.18632/aging.104041)
Supplement: Supplementary Figure 1 [file aging-12-104041-s001..pdf]

SUPPLEMENTARY FIGURE

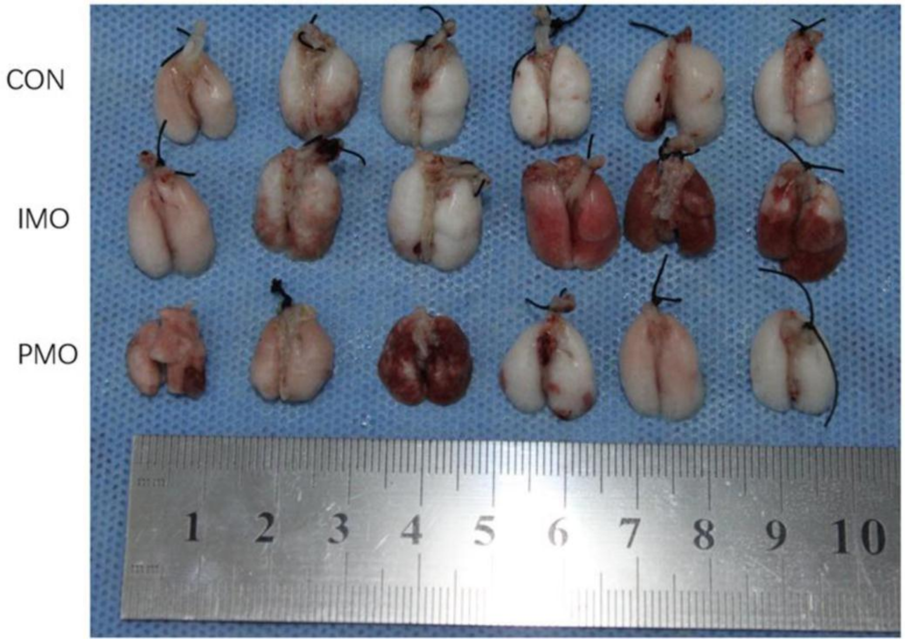

Supplementary Figure 1. All lungs were dissected and then fixed with formalin.
