# Supplementary material for: Patrolling monocytes inhibit osteosarcoma metastasis to the lung
Source: Aging (Albany NY). 2020 Nov 16;12(22):23004–16. doi: 10.18632/aging.104041 (PMC7746373; doi:10.18632/aging.104041)
Supplement: Supplementary Table 1 [file aging-12-104041-s002..pdf]

## SUPPLEMENTARY TABLE

**Supplementary Table 1. The fraction of immune cells.**

| Immune cell type | CIBERSORT fraction in % of all infiltrating immune cells (mean ±SD) | | p-values |
|---|---|---|---|
| | N | M | |
| B cells naïve | 1.15±1.801 | 1.90±2.44 | 0.308 |
| B cells memory | 4.78±4.893 | 4.47±5.276 | 0.674 |
| Plasma cells | 0.31±1.29 | 2.37±5.73 | 0.209 |
| T cells CD8 | 0.47±0.01.45 | 0.96±2.57 | 0.522 |
| T cells CD4 naive | 2.25±0.03.39 | 1.96±3.72 | 0.469 |
| T cells CD4 memory resting | 2.27±0.04.04 | 3.06±5.33 | 0.74 |
| T cells CD4 memory activated | 3.52±0.02.79 | 3.89±2.84 | 0.795 |
| T cells follicular helper | 1.35± 0.01.87 | 1.33±1.53 | 0.914 |
| T cells regulatory (Tregs) | 1.55±0.01.91 | 0.16±0.43 | 0.059 |
| T cells gamma delta | 10.65±5.43 | 9.84±7.83 | 0.573 |
| NK cells resting | 0.62±0.01.27 | 0.89±1.95 | 0.728 |
| NK cells activated | 0.61±0.00.17 | 0.02±0.09 | 0.44 |
| Monocytes | 15.99±0.08.25 | 10.69±6.63 | 0.039 |
| Macrophages M0 | 11.19±0.07.89 | 12.30±8.11 | 0.784 |
| Macrophages M1 | 0.99±0.02.21 | 2.38±4.37 | 0.694 |
| Macrophages M2 | 3.39±0.04.92 | 2.54±3.95 | 0.819 |
| Dendritic cells resting | 2.42±0.03.47 | 1.69±4.13 | 0.719 |
| Dendritic cells activated | 32.15±11.74 | 33.71±11.56 | 0.729 |
| Mast cells resting | 4.28±4.37 | 4.74±5.56 | 1 |
| Mast cells activated | 0.25±0.59 | 0.47±1.16 | 0.34 |
| Eosinophils | 0.47±0.82 | 0.29±0.65 | 0.319 |
| Neutrophils | 0.00±0.00 | 0.30±1.11 | 0.2 |
